# Comparative Proteomics-Based Identification of Genes Associated with Glycopeptide Resistance in Clinically Derived Heterogeneous Vancomycin-Intermediate *Staphylococcus aureus* Strains

**DOI:** 10.1371/journal.pone.0066880

**Published:** 2013-06-28

**Authors:** Hongbin Chen, Yali Liu, Chunjiang Zhao, Di Xiao, Jianzhong Zhang, Feifei Zhang, Minjun Chen, Hui Wang

**Affiliations:** 1 Department of Clinical Laboratory, Peking University People's Hospital, Beijing, People's Republic of China; 2 Department of Clinical Laboratory, Peking Union Medical College Hospital, Beijing, People's Republic of China; 3 National Institute for Communicable Disease Control and Prevention, Chinese Center for Disease Control and Prevention, Beijing, People's Republic of China; Cornell University, United States of America

## Abstract

Heterogeneous vancomycin-intermediate *Staphylococcus aureus* (hVISA) is associated with clinical treatment failure. However, the resistance mechanism of hVISA has not been fully clarified. In the present study, comparative proteomics analysis of two pairs of isogenic vancomycin-susceptible *S. aureus* (VSSA) and hVISA strains isolated from two patients identified five differentially expressed proteins, IsaA, MsrA2, Asp23, GpmA, and AhpC, present in both isolate pairs. All the proteins were up-regulated in the hVISA strains. These proteins were analyzed in six pairs of isogenic VSSA and hVISA strains, and unrelated VSSA (n = 30) and hVISA (n = 24) by real-time quantitative reverse transcriptase–PCR (qRT–PCR). Of the six pairs of isogenic strains, *isaA*, *msrA2* and *ahpC* were up-regulated in all six hVISA strains; whereas *asp23* and *gpmA* were up-regulated in five hVISA strains compared with the VSSA parental strains. In the unrelated strains, statistical analyses showed that only *isaA* was significantly up-regulated in the hVISA strains. Analysis of the five differentially expressed proteins in 15 pairs of persistent VSSA strains by qRT–PCR showed no differences in the expression of the five genes among the persistent strains, suggesting that these genes are not associated with persistence infection. Our results indicate that increased expression of *isaA* may be related to hVISA resistance.

## Introduction

*Staphylococcus aureus* can cause serious hospital- and community-acquired infections, including skin and soft tissue infections, pneumonia, bacteremia, endocarditis, and even septic shock. The high prevalence of methicillin-resistant *S. aureus* (MRSA) and the extensive use of vancomycin have led to the emergence of reduced vancomycin susceptibility among *S. aureus* strains. Heterogeneous vancomycin-intermediate resistant *S. aureus* (hVISA) [vancomycin minimum inhibitory concentration (MIC) ≤2 μg/mL], the precursor of vancomycin-intermediate resistant *S. aureus* (VISA, MIC of 4 − 8 μg/mL), is a strain that contains subpopulations of vancomycin-intermediate daughter cells, but for which the MIC of vancomycin for the parent strain is in the susceptible range. Although vancomycin-resistant *S. aureus* (VRSA) strains are rare, hVISA/VISA are common in the clinical setting, especially in persistent MRSA bacteremia and endocarditis. Our previous studies have shown that the prevalence of hVISA is 13% to 16% in large teaching hospitals in China [1]. Moreover, several studies have indicated that hVISA/VISA infections are associated with vancomycin treatment failure [2], [3].

To date, no specific genetic determinants of hVISA/VISA have been universally defined, whereas VRSA strains acquire the *vanA* gene from *Enterococcus*. Several phenotypic features are characteristic of hVISA/VISA strains, among which significant cell wall thickening is a common feature associated with vancomycin resistance [4]. Compared with vancomycin-susceptible *S. aureus* (VSSA), hVISA produces three to five times the amount of penicillin-binding proteins (PBPs) 2 and 2'. The amounts of intracellular murein monomer precursor in hVISA are three to eight times greater than those in VSSA strains [4]. Factors such as the increased synthesis of non-amidated muropeptides and the resultant reduced peptidoglycan cross-linking contribute to the vancomycin resistance of VISA through increased affinity trapping of vancomycin [5]. In addition to thickened cell walls, hVISA/VISA strains exhibit other phenotypic changes, including reduction in autolytic activity [6], reduced growth rate [7], resistance to lysostaphin [8], PBP changes [9], and metabolic changes [10].

Several transcriptional changes have been detected in hVISA/VISA. DNA microarray analyses have been used to determine changes in the transcriptional profile of hVISA or VISA strains [11]–[15]. However, the protein profiles of hVISA or VISA are rarely analyzed via comparative proteomics. In a proteomics study that compared VSSA and VISA strains, several differentially expressed proteins were identified [16]. Another study identified 65 significant protein abundance changes by comparing three isogenic strains derived from a clinical VISA isolate [17].

To our knowledge, a comparative proteomics analysis of hVISA strains has not been performed to date. Here, we used comparative proteomics to analyze hVISA and VSSA strains isolated from the same patients treated with vancomycin. The differentially expressed proteins identified in our screen were validated in six pairs of isogenic hVISA and VSSA strains and unrelated hVISA (n = 24) and VSSA (n = 30) strains to identify the potential resistance mechanisms of hVISA. To further analyze the potential association of these differentially expressed genes with persistent infection, their expression was examined in 15 pairs of persistent VSSA strains. The results of our study provide insight into the molecular mechanisms underlying hVISA resistance.

## Materials and Methods

The study protocol and written informed consent were approved by the Medical Ethical Committee of Peking University People's Hospital. Written informed consent was obtained from all patients at the time of enrollment.

### Bacterial Isolates

A clinical VSSA (CN9) strain with a vancomycin MIC of 0.5 μg/mL and teicoplanin MIC of 2 μg/mL, and hVISA (CN10) with a vancomycin MIC of 2 μg/mL and teicoplanin MIC of 8 μg/mL were isolated in 2008 from the purulent sputum of an 84-year-old man who had MRSA pneumonia with a 20-year history of emphysema. Strain CN9 (VSSA), isolated after approximately 1 year of hospitalization, was the parental (pretherapy) vancomycin-susceptible isolate; strain CN10 was the hVISA organism recovered during vancomycin treatment. Another clinical VSSA (CN3) strain with vancomycin MIC of 0.5 μg/mL and teicoplanin MIC of 1 μg/mL, and hVISA (CN4) with vancomycin MIC of 0.5 μg/mL and teicoplanin MIC of 2 μg/mL were isolated in 2008 from a 90-year-old woman who had MRSA bacteremia with a 15-year history of diabetes mellitus. Strain CN3 (VSSA), isolated after approximately 1 month of hospitalization, was the parental (pretherapy) vancomycin-susceptible isolate; strain CN4 was the hVISA organism recovered during vancomycin treatment. The above two pairs of VSSA and hVISA strains were selected for comparative proteomics analysis.

Six pairs of VSSA and hVISA strains, and unrelated 24 hVISA and 30 VSSA strains from our previous study were selected to validate the proteomics results [1]. Fifteen pairs of persistent VSSA strains were selected to determine whether the differentially expressed genes were associated with persistent infection. Pairs of bacterial strains with the same genetic background were isolated from the same patient (Table 1). All hVISA strains were confirmed by the population analysis profile (PAP)-area under the curve (AUC) (PAP-AUC) method. All the strains used in the study were tested from frozen stocks and were not frozen and thawed multiple times. Each strain was stored at −80°C in three separate tubes.

**Table 1:** The study isolates, susceptibilities, typing results and clinical aspects.

Isolate a	Specim-en Type	Pheno-type b	MIC (μg/ml) for c:			PAP-AUC	SCC*mec* Typ^b^	Spa Type	MLST	PFGE	Age, years	Diagnosis	Duration of VAN exposure, days d	Therapy e	Outcome
			VAN	TEC	OXA										
Pair 1 (19d)											67	Severe peumonia	6	LEV, TEC	Infection cleared
CN1	sputum	VSSA	0.5	4	>256	0.6	III	t030	ST239	A					
CN2	sputum	hVISA	1	4	256	0.9	III	t030	ST239	A					
Pair 2 (25d)											90	Diabetes mellitus, bacteremia, peumonia	10	MFX, IPM VAN	Infection cleared
CN3	blood	VSSA	0.5	1	256	0.5	III	t030	ST239	B					
CN4	sputum	hVISA	0.5	2	256	0.9	III	t030	ST239	B					
Pair 3 (31d)											54	Coronary heart disease, diabetes mellitus, peumonia	21	IPM, TZP VAN	Infection persisted, patient died of respiratory failure
CN5	sputum	VSSA	1	2	256	0.5	III	t030	ST239	A					
CN6	sputum	hVISA	1	2	256	0.9	III	t030	ST239	A					
Pair 4 (20d)											18	Peumonia, bacteremia	7	LEV, AMK, IPM, VAN	Infection cleared
CN7	blood	VSSA	1	1	256	0.6	III	t030	ST239	C					
CN8	sputum	hVISA	1	2	256	0.9	III	t030	ST239	C					
Pair 5 (95d)											84	Emphysema, peumonia	59	FEP, LEV, IPM, VAN	Infection persisted, patient died of respiratory failure
CN9	sputum	VSSA	0.5	2	256	0.4	III	t030	ST239	B					
CN10	sputum	hVISA	2	8	256	1.0	III	t030	ST239	B					
Pair 6 (29d)											22	Multiple endocrine neoplasia syndrome, abdominal infection	9	CXM, MFX, SCF, VAN	Infection cleared
CN11	blood	VSSA	1	2	>256	0.7	III	t030	ST239	B					
CN12	abdominal fluid	hVISA	1	2	>256	0.9	III	t030	ST239	B					
VSSA-pair1 (45d)											43	Coronary heart disease, peumonia	11	TEC, IPM, SCF, CAZ	Infection cleared
V-F-1	sputum	VSSA	1	2	256	0.7	III	t030	ST239	B					
V-R-1	sputum	VSSA	1	2	256	0.7	III	t030	ST239	B					
VSSA-pair2 (19d)											73	Severe peumonia	16	VAN, LEV, MEM, RIF	Infection cleared
V-F-2	sputum	VSSA	1	2	256	0.7	III	t030	ST239	B					
V-R-2	sputum	VSSA	1	2	256	0.7	III	t030	ST239	B					
VSSA-pair3 (20d)											49	Chronic renal failure, peumonia	6	VAN, CRO, CAZ, LEV	Infection cleared
V-F-3	sputum	VSSA	0.5	2	>256	0.5	II	t002	ST5	D					
V-R-3	sputum	VSSA	0.5	2	>256	0.6	II	t002	ST5	D					
VSSA-pair4 (33d)											43	Systemic lupus erythematosus, skin nd soft tissue infection	16	VAN, CRO, IPM, RIF, SXT	Infection cleared
V-F-4	pus	VSSA	0.5	0.5	0.25	0.3	NA	t034	ST398	E					
V-R-4	pus	VSSA	0.5	0.5	0.25	0.3	NA	t034	ST398	E					
VSSA-pair5 (76d)											66	Chronic obstructive pulmonary disease, peumonia	11	VAN, CRO, SCF, LEV, SXT	Infection cleared
V-F-5	sputum	VSSA	0.5	1	>256	0.5	III	t030	ST239	A					
V-R-5	sputum	VSSA	0.5	1	>256	0.4	III	t030	ST239	A					
VSSA-pair6 (25d)											79	Bronchial asthma, peumonia	9	VAN, CAZ, FEP, AMK, IPM, SCF, LZD	Infection cleared
V-F-6	sputum	VSSA	0.5	2	>256	0.5	III	t030	ST239	B					
V-R-6	sputum	VSSA	0.5	2	>256	0.5	III	t030	ST239	B					
VSSA-pair7 (76d)											79	Chronic obstructive pulmonary disease, peumonia	23	VAN, CRO, LEV, MFX, IPM, TZP	Infection cleared
V-F-7	sputum	VSSA	0.5	1	>256	0.4	II	t002	ST5	D					
V-R-7	sputum	VSSA	0.5	2	>256	0.5	II	t002	ST5	D					
VSSA-pair8 (18d)											84	Chronic obstructive pulmonary disease, peumonia	6	VAN, LZD, IPM, CIP, CAZ, SCF, TZP	Infection cleared
V-F-8	sputum	VSSA	0.5	2	>256	0.6	III	t030	ST239	C					
V-R-8	sputum	VSSA	0.5	2	>256	0.5	III	t030	ST239	C					
VSSA-pair9 (21d)											56	Coronary heart disease, peumonia	18	TEC, IPM, TZP, SCF	Infection cleared
V-F-9	sputum	VSSA	0.5	1	>256	0.6	III	t030	ST239	B					
V-R-9	sputum	VSSA	1	1	>256	0.6	III	t030	ST239	B					
VSSA-pair10 (77d)											75	Chronic obstructive pulmonary disease, peumonia	6	VAN, MFX, TZP	Infection cleared
V-F-10	sputum	VSSA	0.5	2	256	0.5	III	t030	ST239	B					
V-R-10	sputum	VSSA	0.5	2	>256	0.6	III	t030	ST239	B					
VSSA-pair11 (104d)											81	Severe peumonia	52	VAN, IPM, AMK, CAZ, FEP	Infection persisted, patient died of respiratory failure
V-F-11	sputum	VSSA	0.25	2	>256	0.5	III	t030	ST239	A					
V-R-11	sputum	VSSA	0.5	2	>256	0.7	III	t030	ST239	A					
VSSA-pair12 (18d)											43	Coronary heart disease, peumonia	16	TEC, IPM, CAZ, MFX	Infection cleared
V-F-12	sputum	VSSA	0.5	2	256	0.5	III	t030	ST239	C					
V-R-12	sputum	VSSA	0.5	2	256	0.5	III	t030	ST239	C					
VSSA-pair13 (143d)											52	Severe peumonia	18	VAN, SCF	Infection cleared
V-F-13	sputum	VSSA	0.5	1	256	0.5	III	t030	ST239	B					
V-R-13	sputum	VSSA	0.5	2	256	0.7	III	t030	ST239	B					
VSSA-pair14 (15d)											43	Severe peumonia	11	VAN, SCF	Infection cleared
V-F-14	abdominal fluid	VSSA	0.5	2	>256	0.6	III	t030	ST239	A					
V-R-14	abdominal fluid	VSSA	0.5	2	>256	0.6	III	t030	ST239	A					
VSSA-pair15 (20d)											4`2	Severe peumonia	17	VAN, MEM, FEP, LEV, AMK	Infection cleared
V-F-15	sputum	VSSA	0.5	2	>256	0.4	III	t037	ST239	F					
V-R-15	sputum	VSSA	0.5	2	>256	0.6	III	t037	ST239	F					

a interval (day) of the two isolates.

b Defined by population analysis profile (PAP)–area under the curve (AUC) method (PAP-AUC) (see Materials and Methods).

c NA, did not carry SCC*mec*.

d The duration of Vn exposure represents the total length of time that the patient received vancomycin.The VSSA isolate of each pair obtained prior to beginning vancomycin / teicoplanin therapy.

e VAN, vancomycin; TEC, teicoplanin; OXA, oxacillin; LEV, levofloxacin; MFX, moxifloxacin; CIP, ciprofloxacin; IPM, imipenem; MEM, meropenem; AMK, amikacin; FEP, cefepime; CRO, ceftriaxone; CAZ, ceftazidime; CXM, cefuroxime; SCF, cefoperazone-sulbactam; TZP, piperacillin-tazobactam; RIF, rifampin; SXT, sulfamethoxazole-trimethoprim; LZD, linezolid.

### PAP-AUC Method

PAP-AUC was determined as described previously [1]. Briefly, 50 μL of a 0.5 McFarland standard suspension at dilutions of 10^−3^ and 10^−6^ was inoculated onto brain heart infusion (BHI) agar plates containing 0, 0.5, 1.0, 2.0, 2.5, 4.0, and 8.0 μg/mL of vancomycin. After 48 h of incubation at 35°C, the colonies were counted and the log CFU/mL was plotted against vancomycin concentration. The ratio of the AUC of the test isolate to the AUC of *S. aureus* Mu3 was calculated and interpreted as follows: for VSSA, a ratio of <0.9; for hVISA, a ratio of 0.9 to 1.3; and for VISA, a ratio of ≥1.3. *S. aureus* ATCC 29213 was used as the reference VSSA strain.

### Molecular Typing Methods

All isolates were analyzed by SCC*mec* typing, *spa* typing, MLST typing, and PFGE. The SCC*mec* types were determined by the multiplex PCR strategy developed by Kondo et al. [18]. The *spa* typing was performed as described previously [19]. Purified *spa* PCR products were sequenced, and short sequence repeats were assigned by using the spa database website (http://www.ridom.de/spaserver). MLST was carried out as described previously [20]. The sequences of the PCR products were compared with the existing sequences available on the MLST website (http://saureus.mlst.net) for *S. aureus*. DNA extraction and SmaI restriction were performed as described previously [21]. The PFGE patterns were visually examined and interpreted according to the criteria of Tenover et al. [22].

### Protein Sample Preparation

Overnight cultures of VSSA and hVISA strains were diluted at 1/100 in BHI broth and harvested at similar culture densities (exponential phase, OD_600_ nm  = 0.5). The samples were centrifuged at 7,000 g for 10 min to collect the deposits. The deposits were then washed in 50 mM PBS three times and incubated in 220 µL of 20 mM Tris-HCl, pH 7.5; 50 µL of 1 mg/mL lysostaphin; 4 µL of protease inhibitor cocktail; and 6 µL of DNase for 30 min at 37°C. Subsequently, 1.5 mL of 2D lysis buffer (100 mL acetone, 20 mM DTT, 10%TCA) was added, and the samples were vortexed and frozen at –20°C for 2 h. Samples were centrifuged at maximum speed in a microcentrifuge for 2 min to remove insoluble materials, and protein was quantitated using the 2D Quant Kit (GE Healthcare, Arizona, USA).

### Two-Dimensional Gel Electrophoresis (2DE)

2DE was performed as described previously [17]. Samples were run in triplicate. In the first dimension, 500 µg of protein was run on 24 cm Immobiline DryStrips (GE Healthcare) at a pH range of 4 to 7 on an IPGphorII IEF system (GE Healthcare) as recommended by the manufacturer. Strips were equilibrated in equilibration buffer (50 mM Tris-Cl, pH 8.8, 6 M urea, 30% glycerol, 2% SDS, and 0.25% trace of bromophenol blue) containing 10 mg/mL of DTT for 15 min followed by incubation in the same buffer containing 40 mg/mL of iodoacetamide for 15 min. The strips were then applied to 12.5% self-made acrylamide gels using 0.5% agarose in standard Tris-glycine electrophoresis buffer.

Second-dimension sodium dodecyl sulfate–polyacrylamide gel electrophoresis (SDS–PAGE) was performed in a Protean II (Bio-Rad, Hercules, CA, USA) at 40 mA/gel and 15°C until the tracking dye ran-off the gel. Proteins were visualized by Coomassie brilliant blue (CBB) staining. Gels were fixed in 20% TCA for 1 h, stained in 0.1% CBB for 2 h, destained in 40% ethanol and 10% acetic acid for 2×30 min, intensified overnight in 1% acetic acid, and then washed in deionized water for 30 min. Gels were imaged using ImageScanner (GE Healthcare) and images were analyzed by PDQuest 6.0.

### Mass Spectrometry for 2D Gel Protein Identification

Gel plugs containing differentially expressed proteins were excised using a ProXcision robot (Perkin Elmer Inc., Wellesley, MA, US) and subjected to matrix-assisted laser desorption ionization-time of flight/time of flight (MALDI-TOF/TOF) analysis (Bruker Daltonics, Leipzig, GER). Gel plugs were placed in 96-well plates, and then washed with 25 mM NH_4_HCO_3_ (pH 8.0). Gel plugs were pre-frozen at –80°C for 1 h, and then digested with trypsin (Promega, WI, USA). After extraction from the gel into 50% acetonitrile/0.1% formic acid, peptides were lyophilized in a speed vacuum and resuspended in 10 µL of 0.1% formic acid solution. Peptide MS/MS spectra were obtained by MALDI-TOF/TOF (Bruker Daltonics). The resulting MS/MS spectra were interpreted using Mascot and searched against eubacterial proteins in the National Center for Biotechnology Information protein database. Results showing MASCOT score ≥75 and confidence level ≥95% were considered reliable [23].

### Real-Time Quantitative Reverse Transcriptase–PCR (qRT–PCR)

Total RNA was extracted from each sample using the RNeasy Kit (Qiagen, CA, USA). Complementary DNA (cDNA) was generated from total RNA using a random primer hexamer. Gene-specific primers were designed using Primer Express 3.0 (Applied Biosystems) and are shown in Table 2. Samples were run in triplicate and quantified by qRT-PCR following the protocol for SYBR^®^ Premix Ex Taq^TM^ II (TaKaRa, Tokyo, Japan). The mixture was incubated at 95°C for 30 s, and then cycled at 95°C for 5 s and at 60°C for 20 s 40 times using the LightCycler® 480 (Roche, Mannheim, GER). Amplification efficiencies were validated and normalized to the expression of the 16 S rRNA gene as a standard. The quantities of the target and standard genes were calculated according to a standard curve.

**Table 2:** List of primers for real-time quantitative reverse transcriptase– PCR.

Primer Name	Sequence	Length (bp)	NCBI Accession No.
*16S rRNA*-F	GGCAAGCGTTATCCGGAATT	20	NC_002745
*16S rRNA*-R	GTTTCCAATGACCCTCCACG	20	
*isaA*-F	TGGCTCAACGTACTGGTGTT	20	NP_375681.1
*isaA*-R	GACCCCAACCTGGCATAGTT	20	
*msrA2*-F	TGCAACATTAGCAGGAGGATG	21	NP_374474.1
*msrA2*-R	GCATGACCGCCACTATAACC	20	
*asp23*-F	CTTTACGGAAGATTTTAGGTGCTGA	25	NP_375295.1
*asp23*-R	CAAGGTGTATCTGTTGAAGTTGGTG	25	
*gpmA*-F	TCAGACTTTCGGAATAAGGCATC	23	NP_375527.1
*gpmA*-R	ACCTGCTGAAACCGAAGAACA	21	
*ahpC*-F	CACGGCCAATTCCGTCA	17	NP_373615.1
*ahpC*-R	TGACCCATCACAAACAATCACTC	23	

### Statistical Analysis

Statistical analysis was performed using the Wilcoxon rank sum test or One-Way ANOVA test in SPSS (17.0). A *p* value of ≤0.05 was considered statistically significant.

## Results

### Genotypic Characterization of the Isolate Set

Two pairs of isogenic VSSA and hVISA isolates that belonged to SCC*mec*III-ST239-*spa* t030 were selected for the comparative proteomics analysis to minimize variation unrelated to the vancomycin resistance phenotype. Six pairs of isogenic VSSA and hVISA strains, and unrelated VSSA (n = 30) and hVISA (n = 24) strains were selected to validate the differentially expressed genes identified by comparative proteomics screening. Fifteen pairs of persistent VSSA isolates were selected to determine whether the differentially expressed genes were associated with persistent infection.

As shown in Table 1, six pairs of VSSA and hVISA isolates that belonged to the SCC*mec*III-ST239-*spa* t030 type were classified into three PFGE patterns. Of the 15 pairs of persistent VSSA isolates, 11 pairs were SCC*mec*III-ST239-*spa* t030, 2 pairs were SCC*mec*II-ST5-*spa* t002, 1 pair was SCC*mec*III-ST239-*spa* t037, and 1 pair was identified as methicillin-susceptible *S. aureus* (MSSA)-ST398-*spa* t034 type. The 15 pairs of VSSA isolates were classified into 6 PFGE patterns, with each pair of isolates possessing the same PFGE profile.

Of the unrelated VSSA (n = 30) and hVISA (n = 24) strains, 20 VSSA and 8 hVISA strains belonged to the SCC*mec*III-ST239-*spa* t030 type, 5 VSSA and 10 hVISA strains were SCC*mec*III-ST239-*spa* t037, 4 VSSA and 5 hVISA strains were SCC*mec*II-ST5-*spa* t002, and 1 VSSA and 1 hVISA strain belonged to the SCC*mec*IV-ST59-*spa* t437 type.

### Comparative Proteomics Analyses of hVISA and VSSA strains

Five differentially expressed proteins, including probable transglycosylase isaA precursor (IsaA), peptide methionine sulfoxide reductase msrA2 (MsrA2), alkaline shock protein 23 (Asp23), 2,3-bisphosphoglycerate-dependent phosphoglycerate mutase (GpmA), and alkyl hydroperoxide reductase subunit C (AhpC), were identified in two isolate pairs by comparative proteomics (Table 3). These proteins were up-regulated in both hVISA strains, as confirmed by measuring mRNA levels by real-time quantitative reverse transcriptase PCR (Table 4). The differentially expressed proteins belonged to the following categories: (i) defense mechanisms such as MsrA2, Asp23, and AphC; (ii) metabolic functions such as GpmA; and (iii) cell wall biogenesis such as IsaA.

**Table 3:** Proteins differentially expressed between hVISA and VSSA identified in this study.

Accession Number^a^	Protein Name	Gene	Protein PI^b^	Protein MW^c^ (Da)	Protein Score C.I.%^d^	Protein Function/Pathway
Q6GDN1	Probable transglycosylase isaA precursor	*isaA*	6.11	24198.5	100	Cleaves peptidoglycan
Q6GGY3	Peptide methionine sulfoxide reductase 2	*msrA2*	6.64	20545.9	100	A repair enzyme for proteins that have been inactivated by oxidation catalyzes the reversible oxidation–reduction of methionine sulfoxide in proteins to methionine
5HE23	Alkaline shock protein 23	*asp23*	5.13	19179.7	100	Playing a key role in alkaline pH tolerance
Q2YVZ6	2,3-Bisphosphoglycerate-dependent phosphoglycerate mutase	*gpmA*	5.23	26663.5	100	Catalyzing the interconversion of 2-phosphoglycerate and 3-phosphoglycerate/carbohydrate degradation; glycolysis; pyruvate from D-glyceraldehyde 3-phosphate: step 3/5
Q5HIR5	Alkyl hydroperoxide reductase subunit C	*ahpC*	4.88	20963.5	98.842	Directly reducing organic hydroperoxides in its reduced dithiol form

a Accession numbers are from Swiss-Prot or non-redundant NCBI (gi prefix) protein databases.

b Protein PI list experimentally determined isoelectric point values derived from 2-DE spot positions.

c Protein MW list experimentally determined molecular weight values derived from 2-DE spot positions.

d Protein C.I.% indicates confidence interval that the program assigns to the peptide protein matches. >95% score was considered statistically significant.

**Table 4:** **Relative** *isaA*, *msrA2*, *asp23*, *gpmA* and *ahpC* gene expression of hVISA strains compared with that of the parent strains, as determined by quantitative real-time–PCR and normalized to 16S rRNA expression.

Differentially expressed genes	Relative gene expression (arbitrary unit) a						*p-*value b
	CN2/CN1	CN4/CN3	CN6/CN5	CN8/CN7	CN10/CN9	CN12/CN11	
*isaA*	10.2	72.8	674.2	6.7	12.8	5.8	0.028
*msrA2*	1.4	77.2	2.3	1.6	1.2	52.5	0.028
*asp23*	0.6	231.1	44.3	1.7	18.6	11.4	0.075
*gpmA*	0.8	215.7	114.2	13.5	1.8	11.0	0.046
*ahpC*	1.7	12.5	29.0	1.1	1.4	2.7	0.046

a The value of relative gene expression was the averages of triplicate samples.

b *p*-value as determined by Wilcoxon rank sum test.

### Relative Expression of the five Differentially Expressed Proteins in Clinical VSSA and hVISA Isolates

**Figure 1:**
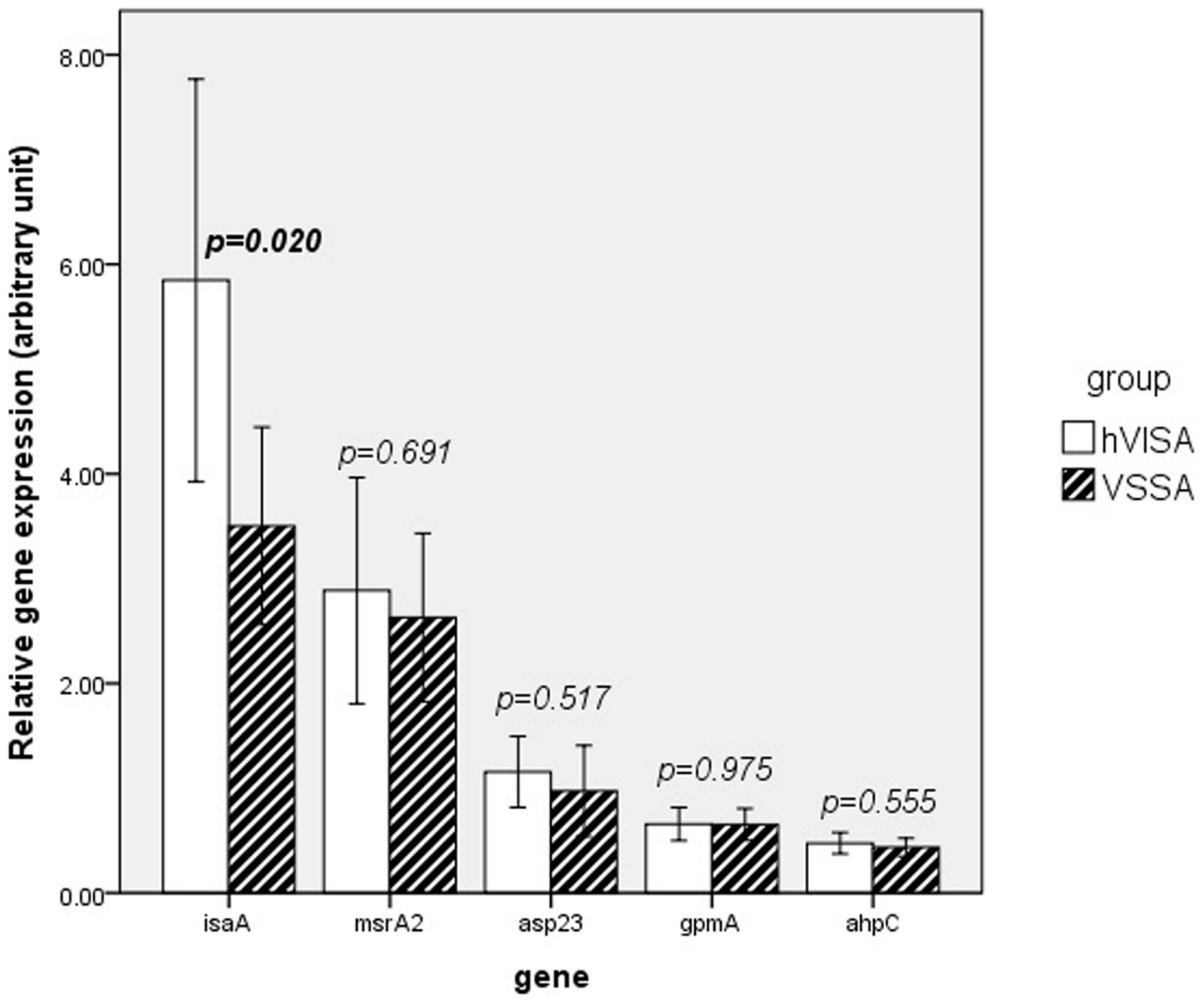
Relative*isaA*, *msrA2*, *asp23*, *gpmA*, and *aphC* gene expression of hVISA strains (n = 24) compared with VSSA (n = 30), as determined by quantitative real-time PCR and normalized to 16S rRNA expression. **Bar means the mean of relative gene expression. Error bar:** **** 95% CI. The value of relative gene expression was the averages of triplicate samples. *p*-value as determined by One-Way ANOVA test.

The expression of the five differentially expressed proteins was assessed in six pairs of isogenic VSSA and hVISA strains by qRT–PCR to validate the accuracy of the results of comparative proteomics. The results showed that *isaA*, *msrA2*, and *ahpC* were up-regulated in all six hVISA strains, whereas *asp23* and *gpmA* were up-regulated in five hVISA strains compared with the VSSA parental strain. The *asp23* and *gpmA* genes were not up-regulated in the CN2/CN1 pair (Table 4). Statistical analysis showed that the expression of these genes, except for *asp23*, was significantly up-regulated in the hVISA strains. The expression of these five genes was also evaluated in unrelated VSSA (n = 30) and hVISA (n = 24) strains by qRT-PCR, which showed that only *isaA* was significantly up-regulated in the hVISA strains (Figure 1).

To determine whether the differentially expressed genes were associated with persistent infection, their expression was assessed in 15 pairs of persistent VSSA strains by qRT-PCR. The results showed no significant differences in the expression level of the five genes among the 15 pairs of persistent VSSA strains (Table 5).

**Table 5:** Relative*isaA*, *msrA2*, *asp23*, *gpmA* and *ahpC* gene expression of persistent *S. aureus* strains, as determined by quantitative real-time–PCR and normalized to 16S rRNA expression.

Isolate	Relative gene expression (arbitrary unit)				
	*isaA* (VSSA-R/VSSA-F) a	*msrA2* (VSSA-R/VSSA-F)	*asp23* (VSSA-R/VSSA-F)	*gpmA* (VSSA-R/VSSA-F)	*ahpC* (VSSA-R/VSSA-F)
VSSA-pair1	2.62	2.18	1.74	1.36	2.48
VSSA-pair2	1.75	0.85	1.03	0.51	1.60
VSSA-pair3	0.87	0.90	0.70	0.31	1.37
VSSA-pair4	1.23	0.95	1.70	0.86	0.91
VSSA-pair5	0.52	0.22	0.22	0.61	0.42
VSSA-pair6	1.65	0.49	0.20	0.65	0.65
VSSA-pair7	1.66	1.64	0.45	0.44	0.42
VSSA-pair8	16.03	1.97	3.21	1.45	1.07
VSSA-pair9	0.27	0.75	0.30	0.31	0.67
VSSA-pair10	0.22	0.91	0.95	0.74	0.79
VSSA-pair11	0.88	0.70	0.86	1.16	1.99
VSSA-pair12	3.53	1.13	0.24	1.40	0.31
VSSA-pair13	0.24	0.83	0.78	0.79	1.15
VSSA-pair14	0.66	0.38	0.93	0.34	0.75
VSSA-pair15	1.65	0.57	0.04	0.19	0.74
*p*-value b	*p* = 1.000	*p* = 0.069	*p* = 0.088	*p* = 0.053	*p* = 0.164

a VSSA-F means vancomycin-susceptible S. aureus (VSSA) isolated from patient prior to vancomycin therapy; VSSA-R means vancomycin-susceptible S. aureus (VSSA) isolated from patient after vancomycin therapy. The value of relative gene expression was the averages of triplicate samples.

b *p*-value as determined by Wilcoxon rank sum test.

## Discussion

Although the clinical importance of hVISA strains has been well-established, the resistance mechanism of hVISA remains unclear. In the present study, the potential mechanism of low-level vancomycin resistance was assessed in two pairs of isogenic VSSA and hVISA isolates by comparative proteomics analysis, which identified five differentially expressed proteins that were up-regulated in both hVISA strains (Table 3).

Among the identified proteins, AphC, Asp23, and MsrA2 are involved in defense mechanisms. AhpC directly reduces organic hydroperoxides to their dithiol forms [24]. The hVISA/VISA strains showed thickened cell walls, increased peptidoglycan crosslinking, and a high positive charge [25], which could have caused changes in oxidation and osmotic pressure inside and outside the cell, and further induced AhpC expression. The stress response gene *asp23*, which encodes the Asp23 protein, is a possible target gene of the key global regulator, SigB [26]. Asp23 has a key role in the alkaline pH tolerance of *S. aureus* [27]. A previous microarray-based study also showed that Asp23 is up-regulated in the hVISA strain [28]. The results of our comparative proteomics analysis showed that MsrA2 was enhanced in both hVISA strains. MsrA2, which catalyzes the reversible oxidation-reduction of methionine sulfoxide to methionine, has a key function as a repair enzyme for proteins inactivated by oxidation. *S. aureus* possesses three MsrA enzymes (MsrA1, MsrA2, MsrA3) [29]. The *msrA* gene is a highly induced member gene of the cell wall stress stimulon (CWSS), which can be induced by cell wall-active antibiotics, such as oxacillin and vancomycin. The up-regulation of *msrA* can lead to an increased rate of peptidoglycan biosynthesis, which results in cell wall thickening [11]. In addition, Msr proteins regulate virulence in several bacteria [29], [30]. In a cDNA microarray study [11], *msrA2* was over-expressed in VISA strains, which coincided with our results. The study also demonstrated that *msrA2* contributes to vancomycin resistance by gene knockout and *trans*-complementation assay [11]. In addition, cell morphology experiments showed that *msrA2* over-expression increases the cell wall thickness of *S. aureus* [11]. Collectively, these observations are consistent with a previous report showing that vancomycin affects the expression of CWSS- associated genes [12].

Another differentially expressed protein, GpmA, functions in cellular metabolism. GpmA catalyzes the interconversion of 2-phosphoglycerate and 3-phosphoglycerate and is therefore involved in the glycolytic pathway [31]. As a key enzyme in glycolysis and energy metabolism, GpmA is a potential target for novel antibiotics [31]. This study is the first to report that GpmA is up-regulated in hVISA.

IsaA, which is involved in cell wall biogenesis, was also over-expressed in both hVISA isolates, as shown in our comparative proteomics results. IsaA cleaves peptidoglycan and thus plays a significant role in peptidoglycan turnover, cell wall crosslinking, and cell division [32]. Therefore, IsaA over-expression could be associated with the thickened cell walls of hVISA strains, which may be related to hVISA resistance. Another comparative proteomics study found that IsaA is up-regulated in the VISA strain Mu50, which is similar to our result [16]. The lack of RNAIII can lead to the over-expression of IsaA [33]. Several studies have indicated that VISA is characterized by *agr* dysfunction or RNAIII down-regulation [6], [34], [35]. A cDNA microarray study showed that IsaA is up-regulated in VRSA strains [36]. Therefore, the *isaA* gene may have an important function in *S. aureus* resistance to vancomycin.

To validate the accuracy of the results of our comparative proteomics analysis, 6 pairs of isogenic VSSA and hVISA strains isolated from the same patient, unrelated VSSA (n = 30) and hVISA (n = 24) strains, and 15 pairs of persistent VSSA strains were selected for confirmation by qRT–PCR. Analysis of the isogenic strains showed that *isaA*, *msrA2*, *gpmA*, and *ahpC* were significantly up-regulated in most of the hVISA strains compared with the VSSA strains, which was partly consistent with the results of comparative proteomics. However, only *isaA* was significantly up-regulated in hVISA strains compared with the unrelated VSSA strains. Therefore, the over-expression of *isaA* may be related to hVISA resistance. Analysis of the 15 pairs of persistent VSSA strains showed no differences in the expression of the identified genes, which indicates that these genes are not associated with persistent infection.

The present study has several limitations. First, the functionality of the identified genes could not be assigned in the absence of gene knockout experiments or further studies. Furthermore, the gene expression changes observed may be a consequence of vancomycin resistance and not causal of this phenotype. For example, these changes may be necessary to compensate for increased cell wall thickness or a consequence of reduced growth rate.

In summary, five differentially expressed proteins, IsaA, MsrA2, Asp23, GpmA, and AphC, were identified in two pairs of isogenic VSSA and hVISA strains via comparative proteomics analysis. The results of qRT-PCR showed that the *isaA* gene was significantly up-regulated in most of the clinical hVISA isolates, suggesting a relation between increased expression of *isaA* and hVISA resistance.
